# Exploring Factors Surrounding Students’ Entrepreneurial Intentions in Medical Informatics: The Theory of Planning Behavior Perspective

**DOI:** 10.3389/fpsyg.2020.544887

**Published:** 2020-10-06

**Authors:** Wen-Hsiung Wu, Chun-Wang Wei, Min-Chun Yu, Hao-Yun Kao

**Affiliations:** ^1^Department of Healthcare Administration and Medical Informatics, Kaohsiung Medical University, Kaohsiung, Taiwan; ^2^Department of Medical Research, Kaohsiung Medical University Hospital, Kaohsiung, Taiwan; ^3^Department of Business Administration, National Kaohsiung University of Science and Technology, Kaohsiung, Taiwan; ^4^Center for Big Data Research, Kaohsiung Medical University, Kaohsiung, Taiwan

**Keywords:** entrepreneurship, entrepreneurial education, medical informatics, theory of planning behavior, intention

## Abstract

The implementation of entrepreneurship and innovation within the health informatics scientific community is comparatively sluggish when compared to other disciplines such as computer science and engineering. The purpose of this paper is to explore the cognitive processes involved in developing intentions to endorse entrepreneurial behaviors via discovering entrepreneurial awareness as a significant influence on an individual’s intentions to identify and adventure market opportunities. In this conceptual paper, insights from Ajzen’s Theory of Planning Behavior (TPB) coupled with self-efficacy beliefs are utilized to develop hypotheses from our research questions. TPB has often been applied to entrepreneurial studies, but for the voluminous body of research devoted to intentions, little has delved into the cognitive processes whereby people develop intentions to entrepreneurial behaviors. Thus, our extended framework can better understand the factors behind entrepreneurial intentions. This research uses a survey tool as a structured questionnaire to explore students’ perceptions of entrepreneurial behavior. The source of the questionnaire is to survey many students from different types of universities in Taiwan. This method allowed respondents to clarify and pose questions. Of the 154 web questionnaires distributed till end of June, 120 were returned, constituting a response rate of 77.9% and Common Method Variance (CMV) had checked. Our results suggest that measuring self-efficacy beliefs in tandem with attitudes toward entrepreneurship provides a better analytical model based on the TPB. The *R*-Square is 41.2% for full model. Moreover, the results help understand entrepreneurial intentions specifically applied to the medical informatics (MI) field which has been under researched. Finally, this study also can guide educators in their efforts to reinforce entrepreneurial behaviors in entrepreneurship education, for example, awareness creation or attractiveness.

## Introduction

Entrepreneurship is seen as an effective means of developing economic benefits from the commercialization of science and technological knowledge (Dodd et al., 2016; Urbano et al., 2019), and has attracted increased academic and policy interest in the past few decades, seeking to use entrepreneurship to accelerate economic development by generating new ideas and turning them into profitable businesses (Turker and Selcuk, 2009). In addition to incubating technical innovation, successful entrepreneurship also provides employment opportunities and increases competitiveness (Asemokha et al., 2019).

Entrepreneurship has played an important role in the progress of modern civilization in almost all periods of human history (Shane and Venkataraman, 2000), and today is a key factor in economic growth, high employment, job creation and positive social development (Sesen, 2013; Gianiodis et al., 2019). In addition, entrepreneurship is widely seen as an effective way to address problems related to unemployment and poverty in the United Kingdom, United States and other countries. As a result, many entrepreneurship education programs have been established to provide effective instruction and mentoring to enhance entrepreneurial intention and skills (Carter et al., 2003; Jennings and Brush, 2013; Yamakawa et al., 2015).

The disciplines of computer science and engineering have led the way in creating a culture of innovation and entrepreneurship, and many successful ventures have started as early collaborations between universities and the business sector. The field of medical informatics (MI) lags behind other disciplines in fostering a culture of innovation and entrepreneurship. As the global economic climate has fluctuated over the past few years, the need for entrepreneurship in health information-related areas has become critical as such activity will help improve medical efficiency and increase employment opportunities for graduates in this field. A recent entrepreneurial trend has emerged in the field of MI, and one of the main focuses of the American Medical Information Association (AMIA) 2015 conference was health information innovation and entrepreneurship (Mcgowan et al., 2012; Househ et al., 2015).

This study examines new definitions of innovation and entrepreneurship as they relate to the MI science community, and discusses various ways to support entrepreneurship in this field. In 2013, the American Medical Association’s (AMA) Medical Education Accelerated Change Conference invited nearly 200 medical education leaders from across the United States to discuss ways to reduce the gap in medical student training and to develop innovation in healthcare systems (Miron-Shatz et al., 2014; Ozdemir et al., 2019). However, this event was marked by the absence of venture capital teams, which could have provided healthcare professionals with important operational training and other healthcare professionals entering the world outside medical school will have an adverse effect.

The early development of entrepreneurship education in Taiwan was dominated by the field of enterprise management, and most entrepreneurship students came from business management backgrounds and lacked diverse practical experience. However, in recent years, in response to social and industrial trends, universities have gradually changed their approach to promote innovation and entrepreneurship, shifting from specialization to cross-domain systematic design and planning, including changes in medical education. In addition, studies in nursing education found that intervention of last mile courses improves professional competence, professional commitment and work pleasure among nursing students (Househ et al., 2015).

In cultivating healthcare professionals, Taiwan’s medical universities attach great importance to the development of skills and knowledge, including the acquisition of professional licenses. Following the reforms in entrepreneurship education, medical universities began to integrate entrepreneurship into its curriculum (Tofighi et al., 2017; Ward et al., 2019). However, universities are responding to changing job market conditions by placing an increasing emphasis on entrepreneurship, but few studies have examined how such courses actually improve learner skills and employability (Wu and Song, 2019). Current course content and design still tends to write start-up proposals, but a lack of funding and access to appropriate technology limit schools in their efforts to teach practical entrepreneurship skills (Hannon, 2013). Scholarly research has found that that the characteristics, abilities, and technological skills of entrepreneurs can be obtained through training and education. Entrepreneurial talent cultivation can thus be regarded as a process of education and training (Kuratko et al., 2004).

Over the past decade, several studies related to entrepreneurship education have examined entrepreneurship training in Taiwan (Chang and Sung, 2009; Chen et al., 2015; Şahin et al., 2019), finding that while people may feel they have the necessary knowledge, skills and ability to start a company, they are reluctant to do so, in part because they considered themselves poorly suited to the challenges inherent in the entrepreneurial process. We believe that a mismatch between personal needs and entrepreneurial content constitutes a gap between self-efficacy and entrepreneurial intention and reduces entrepreneurial intention. In other words, regardless of the level of entrepreneur’s self-efficacy, as long as the entrepreneurial spirit cannot meet his personal needs, individuals will be reluctant to engage in entrepreneurship activity (Hu et al., 2018; Bergner, 2020). There is still considerable controversy as to whether the formation of entrepreneurial intention is worthy of further discussion. The primary concern is that intention may not transfer into start-up behavior, but few studies have addressed this issue. This study seeks to: 1. explore the theoretical basis for building an entrepreneurial curriculum in the field of MI; 2. theoretically explore the behavioral factors that affect student intention to pursue entrepreneurship in this field; 3. suggest a curriculum structure for entrepreneurship education that meets the needs of MI.

## Literature Review

### Entrepreneurship and Entrepreneurship Education

Entrepreneurship cannot be explained by a single theory, and the concept of entrepreneurship has been interpreted differently between different studies, but these typically focuses on several main topics, including entrepreneurship theory, entrepreneur type, entrepreneurial process, organizational form, external environment, and educational outcomes (Westhead et al., 2001). This study adopts a broad, practical rather than theoretical conceptualization of entrepreneurship proposed by the European Commission as follows (European Commission, 2008; Fretschner and Weber, 2013):

“Entrepreneurship refers to an individual’s ability to turn ideas into action. It includes creativity, innovation and risk taking, as well as the ability to plan and manage projects in order to achieve objectives. This supports everyone in day-to-day life at home and in society, makes employees more aware of the context of their work and better able to seize opportunities, and provides a foundation for entrepreneurs establishing a social or commercial activity.”

This definition covers the main theoretical aspects, including the exploration, discovery and exploitation of opportunities by enterprising individuals (Shane and Venkataraman, 2000), the creative breaking of patterns, taking and managing risk, and organizing and coordinating resources (Gibb et al., 2013). In addition, it suggests that entrepreneurship and opportunity development can be carried out within an existing organization as well as through the establishment of a new company (Manimala and Thomas, 2017). Different definitions of entrepreneurship provide for the possibility of new opportunities to develop new businesses and generate economic value within existing organizations (Ireland et al., 2009). In addition, in healthcare research, definitions of entrepreneurship encompass all types of entrepreneurial activities in any area of life and at any scale.

Hansemark (1998) pointed out that entrepreneurship education is seen as a model for changing attitudes and motivations, while traditional education is only seeking to provide change in knowledge and ability (Hansemark, 1998; Fayolle and Gailly, 2015). Other studies have found that, in addition to the obvious advantages of promoting entrepreneurship, entrepreneurship education also has broad market potential (Joensuu-Salo et al., 2015). The ability and desire to start a new business are the two most important prerequisites for success. Not only are entrepreneurial attitudes required in a typical entrepreneurial career, but they play a key role in demanding independent employment relationships (Abualbasal and Badran, 2019). Entrepreneurship education aims to cultivate responsible and enterprising individuals, especially young people, as entrepreneurs or entrepreneurial thinkers who can contribute to economic development and community sustainability.

In 2008, the European Commission proposed a Consortium for Entrepreneurship Education, to encourage creative thinking and develop a strong sense of self-worth and empowerment among young Europeans, stressing the centrality of beliefs, values and attitudes in entrepreneurship education. In addition to business knowledge and skills, the program seeks to enable students to see entrepreneurship as an attractive career option (Holmgren et al., 2004; Raposo and Do Paço, 2011). Jamieson (1984) proposed a three-category framework for organizing entrepreneurial education (Jamieson, 1984). He distinguishes education about entrepreneurship from education for entrepreneurship and in entrepreneurship and the different roles that education plays in developing the relevant skills. The first category is related to enterprise education, and seeks to educate students in all aspects of establishing and operating enterprises from a theoretical perspective, mainly through the cultivation of consciousness. This category includes undergraduate and graduate business courses that seek “to foster skills, attitudes and values appropriate for starting, owning, managing or working in a successful business enterprise.” The author makes distinctions between education about enterprise, for enterprise, and in enterprise to understand the role that different types of education represent (Bae et al., 2014).

### Role of Entrepreneurship Education in Medical Informatics

Developing a model of entrepreneurial management for healthcare organizations requires a discussion of entrepreneurship and its relevance to healthcare (Sharma and Chrisman, 2007; Mitra, 2019). Traditionally, entrepreneurship is studied in the context of startups, family businesses and small and medium sized risk-taking ventures (Guo, 2006). However, for the purposes of this discussion, entrepreneurship in healthcare organizations refers to corporate entrepreneurship, specifically entrepreneurial activities undertaken within an existing organization by individuals (managers) to prompt internal renewal or innovation (Sharma and Chrisman, 2007). Our research argues that entrepreneurship exists in healthcare organizations. In the past, management technology focused on traditional methods, while entrepreneurship focused more on innovative concepts. Managers generate innovative strategies to improve organizational performance through entrepreneurial activity, an essential activity in the turbulent healthcare environment. Entrepreneurial roles can be filled by senior level, operating level and middle level managers (Havinal, 2009). In fact, entrepreneurship can be explained as managers’ innovative and attitudes and creative approaches toward their interaction with the changeable surrounding environment to discover new opportunities (Nobre, 2002). Management is responsible for designing and implementing solutions to problems in organizational settings. Innovative management can use entrepreneurial approaches to face address organizational challenges and in the healthcare context. Based on the above arguments, insight into the healthcare environment limitations and opportunities can be used as a basis for the development of entrepreneurship management education to promote greater creativity and innovation in the organization and operations of healthcare institutions. Healthcare organizations find themselves under increasing financial strain, and an increasing number are turning toward more explicit for-profit business models (Sadler–Smith et al., 2003). Pauly et al. (2002) found that the number of United States-based for-profit managed care organizations (specifically HMOs) increased three times faster than non- profits between 1993 and 1998 (Pauly et al., 2002). In fact, 61% of all-American HMOs are for-profits. This increased market competition is increasingly driving the implementation of entrepreneurship strategies in healthcare organizations. In studying the operations of six teaching hospitals, Guo (2006) found strong evidence of increased entrepreneurship (Guo, 2006).

### Entrepreneurial Behavior and Intention

Theory of Reasoned Action (TRA) is the predecessor of Theory of Planned Behavior (TPB) theory (Madden et al., 1992), and the relationship between the two needs to be discussed to understand the context of TPB’s theoretical development (Sheppard et al., 1988; Mwatsika and Sankhulani, 2016). TRA is founded on the idea that an individual’s behavior is influenced by the attitude and subjective norms of the behavior. This structure is also the basic constructive relationship of TPB. Ajzen (1985, 1991) proposed TPB as a way to increase the predictive ability of TRA, adding Perceived Behavioral Control (PBC) to the original aspects of Attitude and Subjective Norm (Ajzen, 1985, 1991). Cognitive behavior control has become an important core concept of TPB, considering the limitations in real life and improving the deficiencies of the original TRA. Many empirical studies have shown that TPB can indeed improve the interpretation of TRA, and it has been widely adopted in social psychology and other fields (Ajzen, 2005). Mwatsika and Sankhulani (2016) evaluated the influence of entrepreneurship and innovation courses on students’ entrepreneurial orientation. The questionnaire survey deployed in the current study was used to evaluate students’ understanding of entrepreneurship, attitudes toward entrepreneurship, subjective norms, self-efficacy, and entrepreneurial intentions.

Theory of planned behavior proposes three stages of behavior formation: Behavior is determined by an individual’s behavioral intention (Intention), which is subject to the behavioral attitude (Attitude), subjective norms (Subjective Norms), and behavioral control (Perceived Behavior Control). Since Ajzen proposed TPB, PBC has been the spiritual core of TPB, and some subsequent studies have incorporated Self-Efficacy, while others have added Controllability. Some studies have also considered Self-Efficacy as an internal factor and Controllability as an external factor (Terry and O’leary, 1995). For example, self-efficacy is defined by Bandura (1986) as an individual’s ability to complete a particular job (Bandura, 1986), while controllability refers to whether an individual can achieve self-control despite changes to his/her environment (Man et al., 2002).

### Awareness Creation and Attractiveness

To shape entrepreneurial consciousness, universities must systematically promote an entrepreneurial focus (Szczygiel et al., 2014; Amini et al., 2015; Nambisan, 2017). Attention is the first step to creating awareness among students, who can then learn about entrepreneurship in both formal and informal ways. When entrepreneurship is deeply integrated in a university’s culture, the entrepreneurship is reflected in all university activities. The second step is raising student awareness of and interest in entrepreneurship through a series of follow-up activities. Concluding various activities can systematically help students develop a comprehensive interest in and intent to engage in entrepreneurship. Wu et al. (2020) argued to build a sharing platform to increasing attractiveness for integrating service providers and government resources (Wu et al., 2020). The purpose of all these activities is to make entrepreneurship attractive to students, either as something important to their lives and careers, or as a subject of curiosity to better understand before deciding whether it “fits” them (Enninful et al., 2016). In any case, creating awareness among students should lead to the desire to learn more about entrepreneurship through related coursework.

## Methods

### Case Study

This study, conducted a case study as the major methodology to analyses the target cause of a single phenomenon (e.g., An application, a technology, a decision) in an organization over a logical period. The analysis process and theoretical propositions include multiple variables of interest, multiple sources of evidence, and guidance for data collection and analysis (Yin, 2017). Case studies classified by function, including explanatory, exploratory, and descriptive, can be designed around single or multiple cases, and can use qualitative and/or quantitative methods. Qualitative or quantitative evidence is sourced from fieldwork, archival records, verbal reports, observations, or any combination. Our study made related as the available literature offers few courses deployments for entrepreneurship education in the healthcare sector or medical education institutes (Barratt et al., 2011). This study uses multi (embedded) case design focused on Medical Universities in Taiwan. The case study approach involves describing the conditions, rationale, and teaching strategies behind the different structures of entrepreneurship. We first quantified the results of a survey completed by 120 respondents of teachers in medial universities, using a self-structured questionnaire as the main tool for the investigation. For a second qualitative study, we selected 10 administrators or teachers for in-depth interviews. After our initial descriptive analysis is completed, we will perform similar procedures to Yin (2017) pattern matching analysis to identify the context, motivation, and strategy of the link.

### Research Framework

**FIGURE 1:**
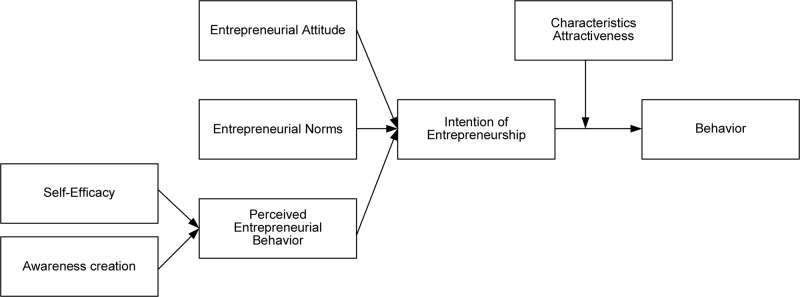
The research framework.

Combining the discussion of the related literature and case study, the research framework is shown in Figure 1. Based on this, we explore theoretically the behavioral factors that affect students in the field to participate in entrepreneurship courses, including how to influence entrepreneurial cognition through self-efficacy and awareness creation, and how to adopt entrepreneurial attitudes, Norms, and cognition further enhances entrepreneurial intentions, and then act, and whether or not they are affected by personal characteristics and attractiveness in the course of taking actions.

### Questionnaire Design

The design of the questionnaire is based on the TPB, and the aspects of discussion include attitudes, subjective norms, perceived behavior control, entrepreneurial willingness, entrepreneurial behavior, and basic information of respondents. Explore the constituent factors and measurement variables of each research facet, and explain the measurement methods. The questionnaire is divided into nine parts. At the same time, in order to avoid questions such as ambiguity in the content of the questionnaire and irregular sentences, which caused the respondents to misunderstand their meanings and answer incorrectly, affecting the validity of the questionnaire, this study commissioned seven people Educate relevant experts and scholars, provide opinions on the content and wording of the questionnaire, and use the Content Validity Ratio (CVR) to quantify the content validity and then modify it to make the questionnaire content more consistent with the theme (Lawshe, 1975). CVR was proposed by Lawshe (1975). The CVR value of this study is 0.83, which exceeds the standard 0.75, and should have expert validity. The questionnaire contents are shown in Table 1.

**TABLE 1:** Construct description and example items.

**Constructs**	**Description**
Attitude	There are 5 items, which are measured by a seven-point scale. Examples of questions: 5 questions that can realize dreams, increase wealth income, and enhance social status.
Subjective norm	There are 5 items in total, measured on a seven-point scale. Examples of question items: 5 items of encouragement from parents, encouragement of trust or worship.
Perceived behavior	There are 5 items, measured on a seven-point scale. Examples of questions: 5 questions about being able to master the funds needed to start a business and having enough confidence to start a business successfully.
Self-efficacy	There are 4 items, measured on a seven-point scale. Question item example: When I decide to start a business, I try to achieve
Awareness creation	There are 4 items, measured on a seven-point scale. Question item example: I think entrepreneurship is very challenging
Intention	There are 4 items, measured on a seven-point scale. Question item example: I am ready to start a business.
Behavior	There are 3 items, measured on a seven-point scale. Question item example: I want to start a business.
Control variables	It including curriculum design, teaching methods, and attractiveness of basic materials
Basic information of respondents:	This section distinguishes entrepreneurial behaviors, all including gender, age, type of school, and schooling system.

This research mainly explores the design of the health information entrepreneurship curriculum from the field characteristics and education model. First, secondary data analysis was used to rectify and analyze the factors related to entrepreneurship in the field of health information, including resources, curriculum design, and teaching models. Then, the focus group method was used to develop the interview structure and questionnaire items. This step will be based on the analysis of the test data. As shown above, a confirmatory factor analysis (CFA) will be performed to confirm whether the results are different from the original research structure. After removing the lower factor load (<0.7), further analysis will be performed. The analysis method will use the Structural Equation Model (SEM) and Partial Least Square (PLS) method to verify the cause and effect relationship, or it can be used as a key factor for success in MI entrepreneurship issues and curriculum development (Chin, 2010). Need analysis and mastery of resources. This study adopts intentional sampling, and surveyed the bachelor’s and senior classes of the bachelor’s class and the master class related to the higher education system, technical and vocational system, and health fields in the south. A total of 154 online questionnaires was sent out, and 120 were returned, constituting a response rate of 77.9% and Common Method Variance (CMV) had checked. The response rate was 77.9%. The CMV has been verified to avoid errors in receiving cases at different periods.

## Results

### Descriptive Analysis

**FIGURE 2:**
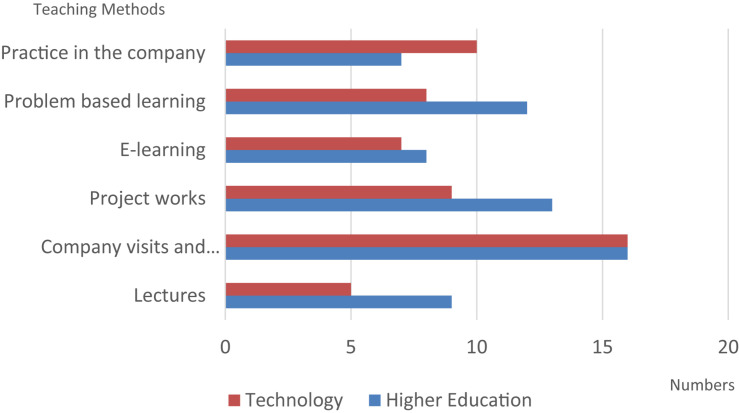
Favorite teaching methods in different type universities.

**FIGURE 3:**
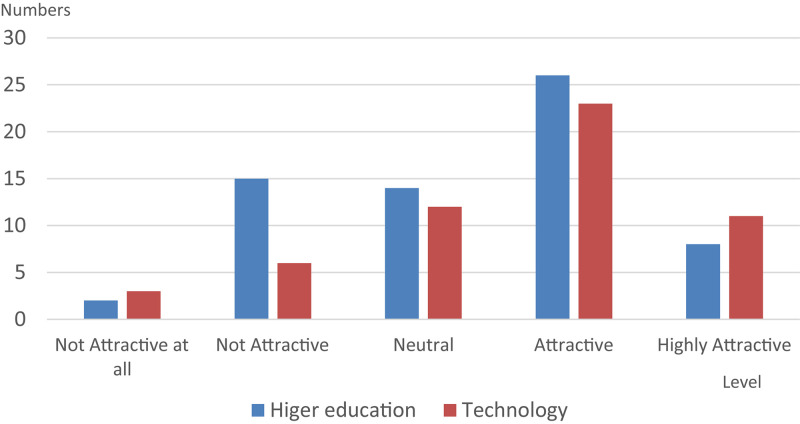
Distribution of attractive level in different universities.

A descriptive statistical analysis was conducted with results shown in Table 2. Nearly 60% of respondents were male and the rate of participating in cross-disciplinary courses was 19.2%. While most of the respondents were undergraduate students, no significant differences were found in terms of level of educational attainment, despite the differences in instructional approaches used in various types of educational institutions. Figure 2 shows the degree of acceptance of the teaching method, and Figure 3 shows the degree to which respondents feel attracted to entrepreneurship as a subject of study. Discussed from the analysis of the trend, the technical and vocational system is relatively attractive compared to the higher education system.

**TABLE 2:** Descriptive analysis of respondents.

Gender	Male	71 (59.2%)
	Female	49 (40.8%)
Joint Program	Yes	23 (19.2%)
	No	97 (80.8%)
University Type	Student Type	*N* = 120 (%)
Higher Education	Graduate	13 (10.8%)
	Undergraduate	52 (43.4%)
Technology	Graduate	7 (5.8%)
	Undergraduate	43 (35.8%)
	Others	5 (4.2%)

### Validity and Reliability of Constructs

Questionnaire items were subjected to average extraction variation of combined questionnaire and validity analysis, using composite reliability (CR) and Cronbach’s α as acceptability criteria. Validation analysis results are presented in Table 3, showing that the seven constructs used in this research all meet the criteria for acceptability, so the questionnaire has reliability and validity. Discriminant validity is used to detect the degree of identification between different constructs between variables. In this study, Chin’s (2010) approach was used to test whether the square root of the average variability extraction amount between each construct exceeded the correlation coefficient value between other constructs. This is different from the traditional SEM which adopts the maxim likelihood method, so there is no statistical result of the goodness of fit (GOF). As shown in Table 4, the questionnaire developed by this research has acceptable discriminative validity. Discriminant validity was also supported because (1) all indicators loaded more strongly on their corresponding construct than on other constructs in the model and (2) the square root of the AVE for each major construct was larger than the inter-construct correlations.

**TABLE 3:** Composite reliability and Cronbach’s α of constructs.

**Constructs**	**Items**	**Composite reliability**	**Cronbach’s α**
Attitude	5	0.820	0.670
Subject Norm	5	0.863	0.801
Perceived behavior	5	0.848	0.856
Self-efficacy	4	0.813	0.807
Awareness creation	4	0.785	0.701
Intention	4	0.744	0.670
Behavior	3	0.759	0.688
			

**TABLE 4:** The average variance extraction of constructs.

**Constructs**	**Composite reliability**	**1**	**2**	**3**	**4**	**5**	**6**	**7**
Attitude	0.820	0.795*						
Subject Norm	0.863	0.093	0.819*					
Perceived behavior	0.848	0.350	0.556	0.812*				
Self-efficacy	0.813	0.378	0.368	0.516	0.773*			
Awareness creation	0.785	0.290	0.482	0.673	0.575	0.741*		
Intention	0.744	0.225	0.465	0.538	0.397	0.603	0.716*	
Behavior	0.759	0.227	0.464	0.495	0.435	0.587	0.655	0.727*

*Diagonal elements are the square roots of average variance extracted (AVE). The square root of the AVE for each major construct was larger than the inter-construct correlations.

### Path Analysis

**FIGURE 4:**
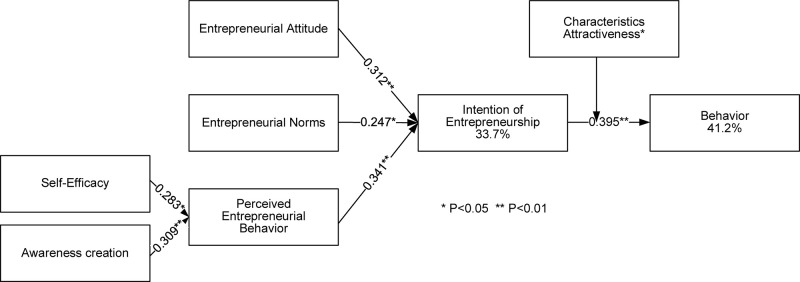
The PLS results.

Path analysis is divided into two phases: measurement model analysis and structural model analysis. The PLS Algorithm is used to detect the reliability and validity of the measurement model, and bootstrapping is performed to generate a Path Coefficient (β value) and *T* value (*t*-value) for statistically significant results. Figure 4 shows the results of PLS pattern analysis, showing the explanatory power of entrepreneurial intention is 33%, and the final explanatory power of entrepreneurial behavior is 40%. Attraction is a moderating variable, and the results show a positive impact on intention and actual behavior. The higher the attractiveness, the stronger the intention to adopt the entrepreneurial behavior.

## Discussion

Taiwan’s educational policies require medical universities to respond to social changes in part by developing skills related to innovation and entrepreneurship in the healthcare sector (Mcgowan et al., 2012). Currently these skills are concentrated in the management and engineering fields, and expanding instruction in these subjects to healthcare requires teachers to develop their own experience and knowledge through engaging in startup activity. Some studies have also suggested that the effectiveness of entrepreneurship teaching activities is positively related to teachers’ previous entrepreneurial experience. Teachers in the healthcare field require sufficient knowledge and ability to start a new business. Therefore, even if their main goal is not to cultivate entrepreneurs, teachers can help increase student motivation and willingness to become entrepreneurs. Therefore, more practical introductory courses in entrepreneurship are needed to stimulate entrepreneurial motivation and behavior (Kuratko et al., 2004). This study finds that, although most of the content of entrepreneurship courses focuses on business ideas and plans, they serve to increase the attractiveness of the subject among students along with their entrepreneurial awareness, thus enhancing their willingness to start a business. Practice-oriented teachers seem willing to support their students in pursuing entrepreneurial careers, possibly due to the teachers’ personal entrepreneurial experience (Bae et al., 2014; Fayolle and Gailly, 2015; Jie and Harms, 2017). The results of this study and past studies have found is consistent, then display the healthcare field has similar circumstances, but then this study to explore the depth of focus and identify influences through survey research. Consequently, we addressed the important findings and compare the results with theory and previous studies on following sections.

From the perspective of students’ entrepreneurial intentions, respondents did not fully realize the goals of entrepreneurial education to become entrepreneurs. Entrepreneurial skills are of particular importance to medical universities who are not on a professional, licensing career track, as such skills will better prepare them to cope with uncertainty and changes in healthcare organizations. These differences may have different utility in the health care field, including the impact on entrepreneurial intentions. In addition, this may also raise new challenges for teachers who will need to create new curricula to teach entrepreneurial courses (Miron-Shatz et al., 2014; Szczygiel et al., 2014).

Appropriate and effective teaching methods for entrepreneurship education also have a critical impact on raising entrepreneurship intention and awareness among students. The teachers in this study used a variety of methods in teaching entrepreneurship, including traditional lectures, but whether such traditional teaching methods can adequately raise entrepreneurial intention and awareness remains doubtful. Therefore, a balance must be struck between different teaching methods to reflect the relationship between the learner goals and the goals of the entrepreneurship plan (Fretschner and Weber, 2013; Miron-Shatz et al., 2014).

Instruction in entrepreneurship skills and abilities should be combined with other teaching activities. *Post hoc* interviews found that healthcare domain teachers who receive more practical teacher training through collaboration with the corporate community will have a better understanding of the nature of entrepreneurship education. In entrepreneurship education, it is particularly important to emphasize the application of teaching methods, reduce the use of traditional teaching models, and adopt more experiential learning methods. These approaches may be more conducive to attracting and engaging students, thereby stimulating their entrepreneurial orientation and intentions (Fayolle and Gailly, 2015; Wu et al., 2020).

The TPB is used as the foundation of behavioral analysis for constructing entrepreneurship courses in the field of health information. Indeed, it has been found that creating awareness among students raises their interest. “You can learn more about entrepreneurship and take one or more related courses in the field of entrepreneurship, and how the design of the course will induce students’ intentions and behaviors is an issue that can be explored.” The questionnaire used here covers attitudes, subjective norms, perceived behavior control, entrepreneurial willingness, and entrepreneurial behavior, and its good reliability and theoretical foundation make it suitable for exploring the behavioral factors that affect students’ participation in entrepreneurship courses in the healthcare field (Hu et al., 2018; Atitsogbe et al., 2019). Although analysis of control variables does not reveal a moderating effect, further *post hoc* interviews can be used to clarify whether it is appropriate to discuss entrepreneurial issues.

Different teaching methods can be used to appropriately serve students of different ability levels and promote entrepreneurship intention. A curriculum structure for entrepreneurship education that meets MI needs should including guidance to raise student awareness of “entrepreneurship,” thereby raising interest and intention to approach organizational and operational problems with greater creativity. After achieving awareness, students can make informed course selection decisions in the field of entrepreneurship and better understand the role such courses and other activities serve in developing entrepreneurship as a future career path (Atitsogbe et al., 2019; Şahin et al., 2019; Wu and Song, 2019).

## Conclusion

The results of this study found that the entrepreneurial education provided by Taiwan’s medical universities does not match recommended best practices, mainly because most teachers have little or no opportunity to practice or experience entrepreneurship, and the curriculum should be modified to focus more on the transfer of entrepreneurship knowledge along with hands-on experience in implementing best practices. Of course, we are limited to the characteristics of the study universities in Taiwan as well as having different than the other countries. In practice, entrepreneurial awareness can be raised through teaching examples and inviting entrepreneurs as guest lecturers. The academic community should also consider how to best teach relevant theory and cases to supplement the practical learning experience, increase subject attractiveness, raise subject awareness, and promote self-efficacy to better encourage entrepreneurial intentions and behaviors. Our preliminary results outline several success factors to improve the implementation of entrepreneurship education within medical universities: (1) incorporate innovation, entrepreneurship, and practice; (2) create strong links to industry and healthcare organizations; (3) provide support and resources for entrepreneurship education; (4) encourage a culture of innovation and entrepreneurship; (5) develop policies that support innovation and entrepreneurship based on medical/healthcare perspectives. By implementing these strategies, we predict that entrepreneurship education may be more easily applied to East Asian/SE Asian medical universities.

## Data Availability Statement

The raw data supporting the conclusions of this article will be made available by the authors, without undue reservation.

## Ethics Statement

Since this study is a teaching evaluation derived from the teaching process, the student did not lose any rights because of this research, and we did not disclose any student’s personal information in the manuscript. Students are already adults. Oral informed consent was obtained from all participants. For the reported study, no ethics approval was required according to the guidelines of the Kaohsiung Medical University or Ministry of Health and Welfare of Taiwan.

## Author Contributions

H-YK and C-WW: supervision of the project, design of the research, organization of the experiment conduction, data analysis and interpretation, writing and revision of the article. M-CY and W-HW: organization of the experiment conduction, data analysis and interpretation, and writing of the article. All authors contributed to the article and approved the submitted version.

## Conflict of Interest

The authors declare that the research was conducted in the absence of any commercial or financial relationships that could be construed as a potential conflict of interest.
